# The Effect of Zeaxanthin on the Visual Acuity of Zebrafish

**DOI:** 10.1371/journal.pone.0135211

**Published:** 2015-08-12

**Authors:** Eric A. Saidi, Pinakin Gunvant Davey, D. Joshua Cameron

**Affiliations:** 1 College of Optometry, Western University of Health Sciences, Pomona, CA, United States of America; 2 Graduate College of Biomedical Sciences, Western University of Health Sciences, Pomona, CA, United States of America; Harvard University, UNITED STATES

## Abstract

Oral supplementation of carotenoids such as zeaxanthin or lutein which naturally occur in human retina have been shown to improve vision and prevent progression of damage to advanced AMD in some studies. The zebrafish eye shares many physiological similarities with the human eye and is increasingly being used as model for vision research. We hypothesized that injection of zeaxanthin into the zebrafish eye would improve the visual acuity of the zebrafish over time. Visual acuity, calculated in cycles per degree, was measured in adult zebrafish to establish a consistent baseline using the optokinetic response. Zeaxanthin dissolved into phosphate buffered saline (PBS) or PBS only was injected into the anterior chamber of the right and left eyes of the Zebrafish. Visual acuities were measured at 1 week and 3, 8 and 12 weeks post-injection to compare to baseline values. Repeated measures ANOVA was used to compare visual acuities between fish injected with PBS and zeaxanthin. A significant improvement in visual acuity, 14% better than before the injection (baseline levels), was observed one week after injection with zeaxanthin (p = 0.04). This improvement peaked at more than 30% for some fish a few weeks after the injection and improvement in vision persisted at 3 weeks after injection (p = 0.006). The enhanced visual function was not significantly better than baseline at 8 weeks (p = 0.19) and returned to baseline levels 12 weeks after the initial injection (p = 0.50). Zeaxanthin can improve visual acuity in zebrafish eyes. Further studies are required to develop a better understanding of the role zeaxanthin and other carotenoids play during normal visual function.

## Introduction

The macula is the center of the retina responsible for detailed central vision and houses three principle carotenoid pigments: lutein, zeaxanthin, and meso-zeaxanthin, which are collectively referred to as retinal or macular pigment.[1, 2] Zeaxanthin is one of the most common carotenoids found in nature and can be found in many commonly eaten vegetables.[3] Zeaxanthin’s pre-receptoral filtration of blue light is believed to reduce the adverse impact of optical limitations such as glare disability, light scatter, and chromatic aberration, thereby optimizing contrast sensitivity and visual acuity in humans.[4] It follows, therefore, that augmentation of macular pigments, such as zeaxanthin, would result in enhanced contrast sensitivity and improved visual acuity. It is shown by clinical trials in humans that macular pigments are necessary for optimal visual function, including visual acuity in both diseased and non-diseased eyes, and this can be achieved by supplementation with a formulation containing the constituent carotenoids (lutein, zeaxanthin, and meso-zeaxanthin).[5–7] The level to which visual function may be enhanced has varied between studies warranting continued investigation into the role carotenoid supplementation plays in vision. [8–12]

Zebrafish (*Danio rerio*) are a proven model for vision research due to the physiological similarity of their eye to other vertebrates; however, the methods to measure specific visual responses in adult zebrafish are newly developing.[13] We previously developed a method to repeatedly and accurately utilize the optokinetic response (OKR) to measure and calculate visual acuity in adult zebrafish.[14] We hypothesized that a direct intracameral injection of zeaxanthin in anterior chamber will improve the visual acuity of the zebrafish over time. In this study, we injected zeaxanthin into the eyes of adult zebrafish and measured their visual acuity to observe whether zeaxanthin alone has a beneficial effect.

## Methods

### Zebrafish Optokinetic Response

AB zebrafish are maintained under standard conditions: 28.5˚C on a 10 hour dark/14 hour light cycle. [15] All animal protocols adhere to the ARVO Statement for the Use of Animals in Ophthalmic and Vision Research and were approved by the Institutional Animal Care and Use Committee at the Western University of Health Sciences. Zebrafish were selected based on age (1.5–3yrs) and absence of obvious ocular abnormalities such as cataract. The optokinetic response (OKR) and subsequent visual acuity calculation are done as previously published.[14] Briefly, OKR analysis begins by anesthetizing a fish in 0.016% tricaine for 2–3 minutes. The fish is then placed on a small Styrofoam platform with the eyes and gills suspended over the edge. To keep the fish immobilized while also avoiding injury, a thin paper towel is placed over the body of the fish and 2–3 pieces of foam are pinned over the fish.

**Fig 1:**
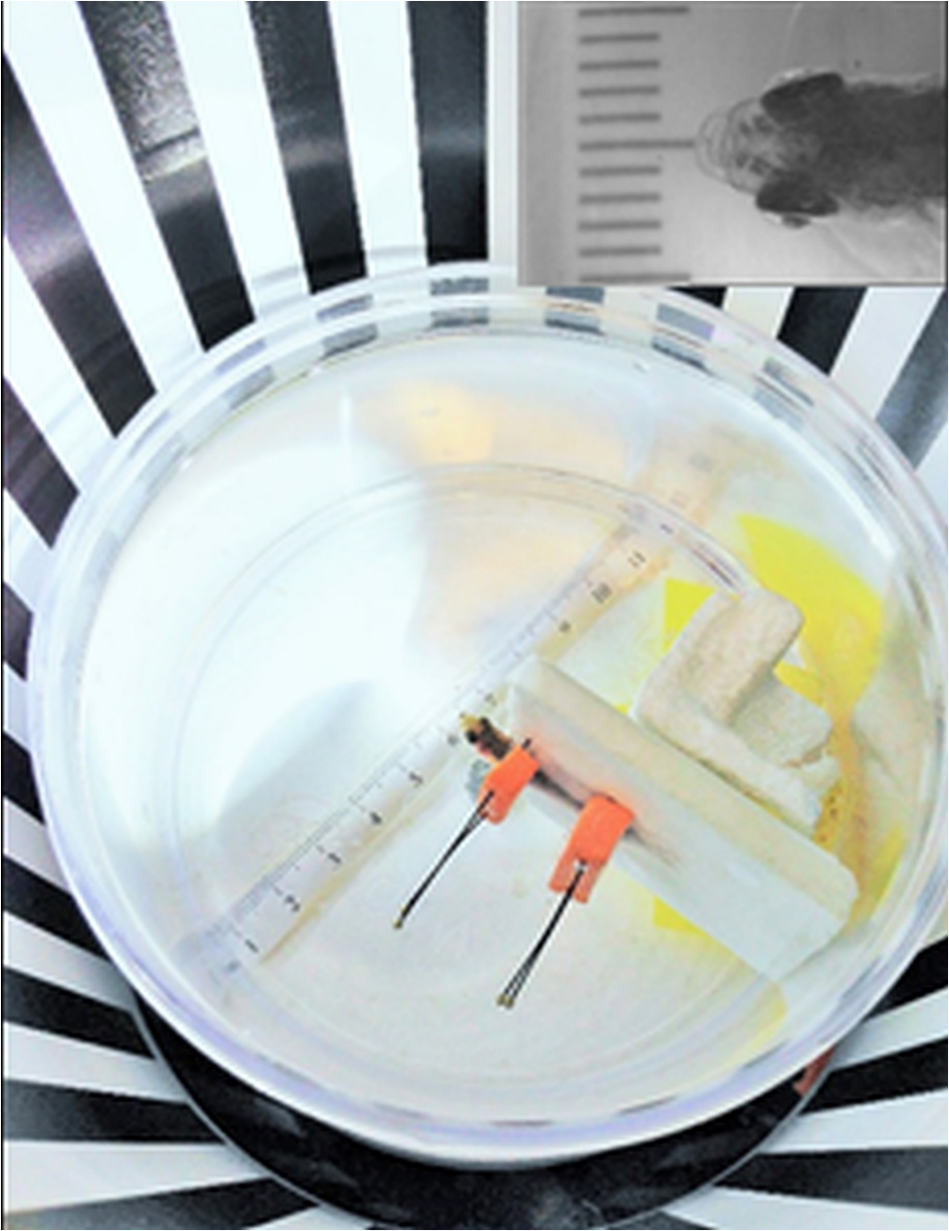
Positioning zebrafish in the OKR device. The fish is positioned upright with the eyes approximately 7.3cm from the edge of the drum (inset image). The fish is given time to recover from anesthesia while positioned within the cylindrical tank of water.

The OKR device consists of a 14.5cm diameter rotating drum and microscope with the light intensity kept at 5140lux and rotation at 8–12rpm. A camera provides a live feed on an adjacent monitor. All OKR responses are recorded digitally in using a video recorder for independent post hoc observation. The fish is placed inside a cylindrical water filled tank that fits inside the rotating drum (Fig 1). The identification of animals either control or experimental group were masked from the observer (EAS) during the OKR. Once an initial OKR was taken, the fish were kept in individual tanks to allow for monitoring of each fish’s visual changes over time.

### Visual Acuity

**Fig 2:**
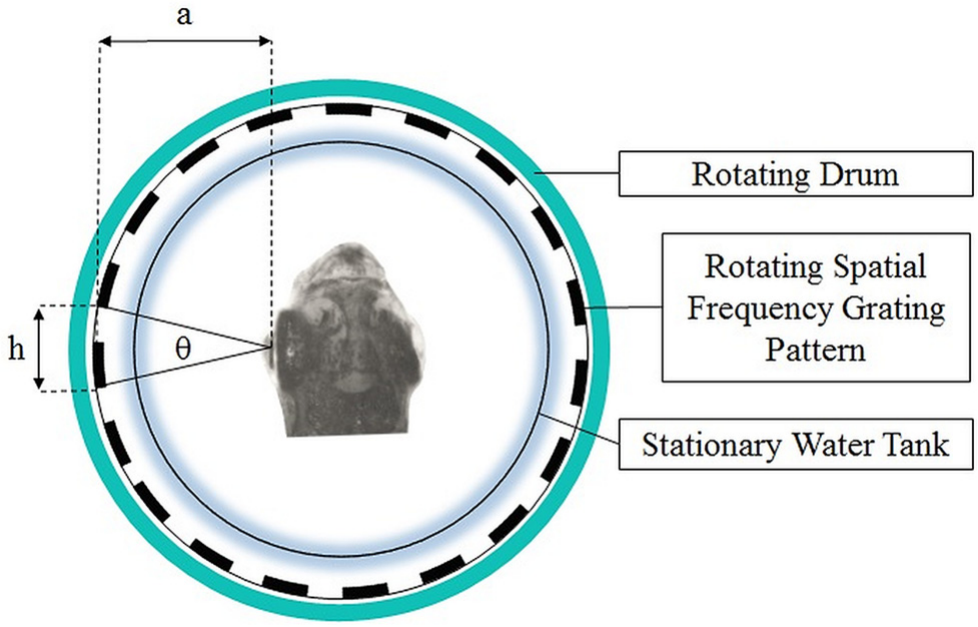
Schematic for calculating cpd. The head in this figure is enlarged for demonstration purposes.

The binocular spatial acuity thresholds are determined using different sized gratings at 100% contrast, replacing each grating with a smaller grating each time. Gratings ranged in size from 0.72mm to 1cm with ~0.05mm increments up to 2mm and 1mm increments thereafter. This process was repeated until an OKR could no longer be elicited using a modified staircase approach as described previously.[14] The size of the smallest grating that caused an OKR was used to calculate the visual acuity for the fish. To determine visual acuity, cycles per degree (cpd) is calculated using the following formula: ½[arctan (*h*/2*a*)] where *a* is the distance from the center of the lens to the grating and *h* is the length of one cycle of the smallest grating at which OKR was observed (Fig 2). Visual acuities were measured at baseline and 1 week and 3, 8 and 12 weeks post-injection.

### Eye Injection

**Fig 3:**
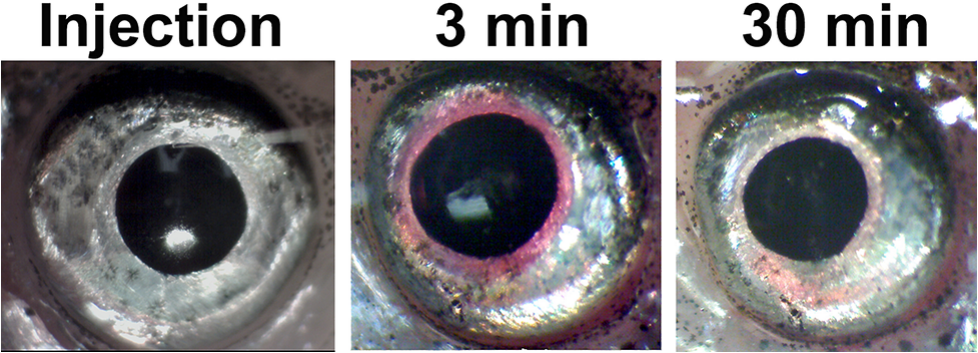
Injection of PBS with 0.001% Phenol Red into the Zebrafish Eye. From left to right, just prior to the time of injection the eye is clear. Once both eyes have been injected (a period of about 3 minutes), the dye is visible in the anterior chamber. After 30 minutes the dye has been pumped to the vitreous via the normal aqueous flow dynamics of the zebrafish eye.

The zebrafish were divided into 3 groups of 10 to 18 fish. A volume of 9.2nL of zeaxanthin (ZeaVision Chesterfiefld MO, USa–C) with a final concentration of 0.46ng/uL was injected into the anterior chamber of both eyes in one group of 11 zebrafish using a Drummond Nannoject II.[16] Another group was injected similarly with 9.2nL of phosphate buffered saline (PBS) with 0.001% phenol red–the solution that zeaxanthin was dissolved in (n = 10). An additional group of 18 fish were used as a control receiving no eye injections. All injections were performed by a single trained investigator (DJC). Fig 3 shows the eye of the fish immediately after injection. The time taken to perform injections on both eyes was 3 minutes on average and the fish were put into their individual tanks after injections were completed.

### Statistical analysis

Repeated measures ANOVA was used to compare the change in VA within each group after baseline acuity measurement and an injection of either PBS or zeaxanthin using SPSS v14. Pairwise comparisons of the means were corrected using a Bonferroni correction for multiple comparisons and significance was set to α = 0.05. Normalized visual acuity values are presented as a percent difference relative to an averaged baseline value taken from all fish at the start of the experiment.

## Results

**Fig 4:**
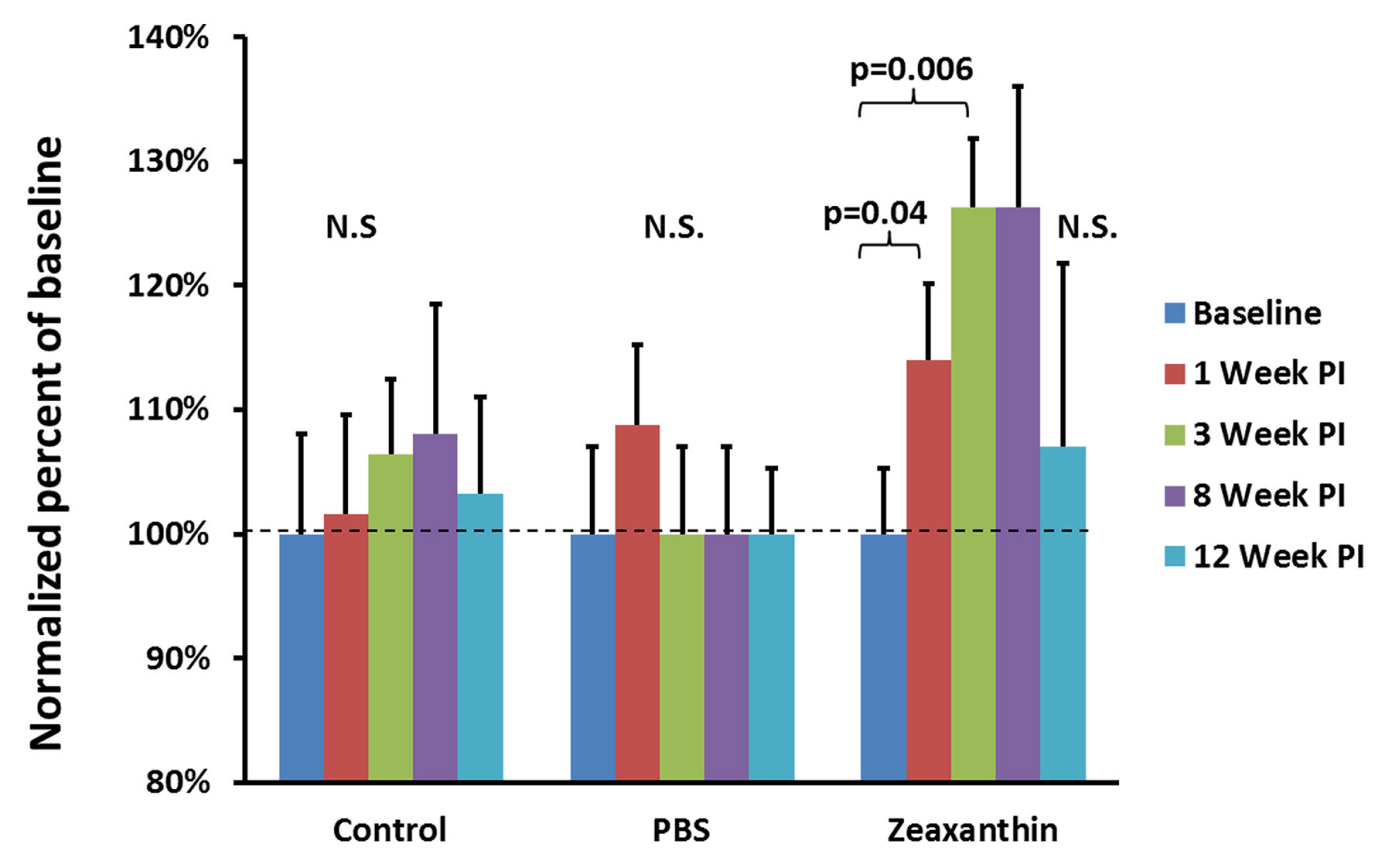
Normalized visual acuity measurements represented as a percent of baseline after injections. The values presented are mean percentages normalized to baseline means for each group. Control fish (n = 17) did not receive an injection and their visual acuity did not significantly (N.S.) change. The PBS group (n = 13) received an equivalent volume of PBS injected into their eyes and did not show a significant change compared to baseline post injection (PI). The group injected with zeaxanthin (n = 13) had an increased visual acuity at 1 and 3 weeks PI and then saw a return to approximate baseline acuity levels by 12 weeks. Error bars represent normalized standard errors. The dotted line identifies the normalized starting visual acuity for each group.

Table 1 shows the visual acuities and the standard deviations of the different groups tested. The baseline visual acuity of all fish at the beginning of the experiment was 0.59 ± 0.17 cpd. The average visual acuity of zebrafish injected with zeaxanthin significantly increased approximately 14% after just 1 week following an injection of zeaxanthin: (0.65 ± 0.15cpd; p = 0.04). (Fig 4) Two weeks later, 3 weeks after the injections, the zeaxanthin injected fish continued to have elevated visual acuities peaking at approximately a 26% increase over baseline (0.72 ± 0.15cpd; p = 0.006). (Fig 4) Neither the fish injected with PBS nor the control group without an injection showed a significant improvement in visual acuity at week 1 and week 3 from baseline (1 week PBS 0.61 ± 0.15cpd and control 0.63 ± 0.18cpd; p = 0.39 and 0.50 respectively; 3 weeks PBS 0.57 ± 0.14cpd and control 0.66 ± 0.17cpd; p = 0.50 and 0.50 respectively.

**Table 1:** Visual acuities of the groups of fish at each time point. Group means are presented with corresponding standard deviations of the mean. Bold values represent statistically significant improvements relative to the baseline using pairwise comparisons after a Bonferroni correction using repeated measures ANOVA. Significance was set at ɑ = 0.05. PI–post injection.

	N	Baseline	1 Week PI	Sig (ɑ)	3 Week PI	Sig (ɑ)	8 Week PI	Sig (ɑ)	12 Week PI	Sig (ɑ)
**Control**	17	0.62 ± 0.20	0.63 ± 0.18	0.50	0.66 ± 0.17	0.50	0.67 ± 0.16	0.23	0.64 ± 0.12	0.50
**PBS**	13	0.57 ± 0.15	0.62 ± 0.15	0.39	0.57 ± 0.14	0.50	0.57 ± 0.11	0.50	0.57 ± 0.09	0.50
**Zeaxanthin**	13	0.57 ± 0.12	**0.65 ± 0.15**	**0.04**	**0.72 ± 0.16**	**0.006**	0.72 ± 0.20	0.19	0.61 ± 0.18	0.50

Eight weeks after being injected with zeaxanthin fish began to show a decline in visual acuity such that their average acuity was no longer statistically significant from baseline (0.72 ± 0.20cpd; p = 0.19). A month later at the 12 weeks post injection time point, the zeaxanthin injected fish visual acuity was within 5% of its original baseline (0.61 ± 0.18cpd; p = 0.50). The visual acuities for neither the group injected with PBS nor the control group significantly changed at the 8 or 12 weeks after the injection timepoints (8 weeks PBS 0.57 ± 0.11cpd and control 0.67 ± 0.16cpd; p = 0.50 and 0.23 respectively; 12 weeks PBS 0.57 ± 0.09cpd and control 0.64 ± 0.12cpd; p = 0.50 and 0.50 respectively). (Fig 4)

## Discussion

This study demonstrated a significant improvement in visual acuity of zebrafish whose eyes were injected with zeaxanthin. Over the course of the experiment, the PBS and control groups had small changes in their visual acuities, peaking at a 12% change relative to baseline, compared to the 26% improvement seen with zeaxanthin. The variation seen in the group injected with PBS is similar to uninjected control visual acuities measured over the same course of time.[14] All groups showed some variability in acuity as measured using OKR, however the OKR change in the group injected with zeaxanthin was more than double compared to the group with PBS injections and the control group.

Zebrafish do not have a macula identical of that of humans.[17] It is seen in prior reports that when dye labeled injections are performed in the anterior chamber of the fish the colored dye combines with intraocular fluids of the fish and the “intensity of color” declines over time as this fluid is drained through aqueous outflow pathways.[16] Prior reports have also shown that the drainage of aqueous humor is behind the fish iris and when vasoproliferative substances are injected the fish retina does show an increased vasoproliferation.[16, 18] Although the evidence of true retinal uptake is not available it is very likely the zeaxanthin deposits to some degree in the fish retina thus leading to an increased visual performance and overtime as might be expected, the effect of zeaxanthin decreased many weeks after the injection as the visual acuity returned to baseline levels. Although OKR was a valuable device to measure visual acuity, it is also possible to use other methods of assessing visual function in the zebrafish. It will also be interesting to see if the visual performance in terms of other tests, for example electroretinography (ERG) or contrast sensitivity, show improvement after zeaxanthin injections and if this translates to cellular protection as evaluated by histology. [19–21]

There is evidence that macular pigment carotenoids, such as zeaxanthin, are necessary for optimal visual function and can aid in the prevention of AMD.[22–25] Clinical studies that evaluated the efficacy of oral supplementation of carotenoids found that the serum concentration of carotenoids can be increased with oral supplementation however the degree of augmentation and associated benefits was variable.[8–12] The results of this study indicate that the delivery method may have played a role in part for the variable outcomes. All fish that had a zeaxanthin injection showed improvement in vision and thus further benefits in humans may be possible if intraocular injection delivery route is utilized instead of oral route. Zebrafish have a unique aqueous humor outflow pathway that enabled corneal injection, whereas direct vitreous intravitreal injections may be more appropriate in other animals or humans.[16, 26]

Intraocular injection of zeaxanthin in its pure form enhanced visual performance as measured using OKR in this study. This may in part be due to decreasing glare and absorption of stray light, specifically blue light, therefore improving retinal sensitivity.[27] Because the zebrafish have a regular array of blue light sensitive cones, the effect may be enhanced in zebrafish[28] The observed visual improvement seems to persist 3weeks post injection. This may have implications in various diseases, particularly some grades of non-exudative age related macular degeneration that show little to no improvement to conventional forms of antioxidant supplementation (AREDS 1 and 2).[29, 30] In cell culture studies zeaxanthin and, in some cases, other carotenoids have been shown to reduce VEGF secretion and protect cells from oxidative stress.[31–33] If zeaxanthin could have a similar effect in vivo, then it may have implications for the treatment of exudative macular degeneration as well.

The present study used 46ng/μl of zeaxanthin given in a single injection. At this dose it appeared to be safe with none of the fish showing any adverse effects. Future experiments are needed to look at the effect of doing multiple injections with possibly increased doses over a long time-course and to determine the uptake of zeaxanthin in the retina. Both of these avenues will provide details as to the efficacy and maximum effect in terms of visual performance that can be obtained. It will also be interesting to see if other carotenoids, such as lutein, can independently or in combination with zeaxanthin provide a similar improvement in acuity. Further, it will be interesting if combinations of various ratios of these carotenoids can be beneficial as the levels of zeaxanthin versus lutein is 2:1 in the macula.[2, 34]
